# Assessment of Apparent Diffusion Coefficient and Perfusion Values of the Placenta in Intrauterine Growth Restriction by Using 3Tesla Magnetic Resonance Imaging (MRI) in an Indian Population: A Pilot Study

**DOI:** 10.4314/ejhs.v36i1.6

**Published:** 2026-01

**Authors:** Priyanka Chandra Sekhar, DSJ Chaitanya Deep

**Affiliations:** 1 Imaging Technology, Division of Allied Health Sciences, School of Health Sciences, The Apollo University, Murukambattu, Chittoor; 2 Department of Radiology, Sri Ramachandra Institute of Higher Education and Research, Chennai, Tamil Nadu 600116, India; 3 Department of Radiology and Imaging Sciences, Sri Venkateswara Medical College, SVRRGGH/RUIA. Tirupati

**Keywords:** Intrauterine Growth Restriction, pseudo-continuous arterial spin labeling, ADC, perfusion

## Abstract

**Background:**

Intrauterine growth restriction (IUGR) is defined as an estimated fetal weight below the 10th percentile for gestational age and is commonly associated with placental insufficiency and abnormal fetoplacental oxygenation. This study aimed to assess placental apparent diffusion coefficient (ADC) and perfusion values in IUGR using 3T magnetic resonance imaging (MRI).

**Methods:**

Sixty pregnant women (30 with IUGR and 30 controls; gestational age 20–38 weeks) underwent placental MRI on a 3T system. The imaging protocol included T2-weighted anatomical sequences, diffusion-weighted imaging (b-values: 0 and 700 s/mm^2^; NEX = 3), and three-dimensional pseudo-continuous arterial spin labeling (3D pCASL) perfusion imaging (TR/TE = 5000/50 ms, labeling duration = 1500 ms, post-labeling delay = 1525 ms, 30 label/control pairs). ADC and perfusion maps were generated, and multiple elliptical regions of interest (ROIs; 40–60 mm^2^) were placed throughout the placenta. Mean values were calculated for each subject.

**Results:**

Mean placental perfusion was significantly lower in the IUGR group compared with controls (102.5 ± 18.7 vs. 120.2 ± 23.7 ml/100 g/min, p = 0.002). ADC values were also significantly reduced in IUGR placentas (1.83 ± 0.10 × 10^−3^ mm^2^/s vs. 2.02 ± 0.10 × 10^−3^ mm^2^/s, p = 0.001). Receiver operating characteristic (ROC) analysis demonstrated fair diagnostic performance for perfusion (AUC = 0.703) and excellent performance for ADC (AUC = 0.919).

**Conclusion:**

Both placental ADC and perfusion values were reduced in IUGR. However, because ADC is influenced by perfusion bias, the most clinically relevant finding is the direct quantification of reduced placental perfusion using 3D pCASL at 3T. This technique provides absolute perfusion values in physiological units and may serve as a valuable non-invasive biomarker of placental dysfunction in IUGR

## Introduction

Intrauterine growth restriction (IUGR) is defined as an estimated fetal weight below the 10th percentile for gestational age according to the American College of Obstetricians and Gynecologists and is frequently associated with placental insufficiency and abnormal fetoplacental oxygenation (1,2). Fetuses with severe IUGR experience chronic hypoxia and are at increased risk of preterm birth and acute perinatal compromise. Late-onset IUGR has also been linked to adverse long-term outcomes, including suboptimal academic performance and impairments in attention and cognitive function (3).

The global incidence of IUGR is estimated to be approximately 24%, with Asia accounting for nearly 75% of affected infants. After prematurity, IUGR remains a leading cause of perinatal morbidity and mortality. Consequently, early and accurate assessment of placental function is essential for the effective management of pregnancies complicated by IUGR (4).

Ultrasonography (US) and magnetic resonance imaging (MRI) are well-established modalities for evaluating fetal anomalies (5). According to guidelines from the American College of Radiology and the American College of Obstetricians and Gynecologists, MRI performed at field strengths of 3.0 T or lower has not been associated with adverse fetal effects; nevertheless, its use should be judicious throughout pregnancy (6,7). Fetal MRI serves as an important complementary tool to US for the detection of both morphological and functional abnormalities (8).

MRI relies on magnetic fields and radiofrequency pulses, involves no ionizing radiation, and is considered safe during pregnancy. Advanced diffusion- and perfusion-weighted MRI techniques provide valuable functional information regarding placental microstructure and hemodynamics. The term perfusion refers to blood flow at the capillary level and is expressed in units of ml/min/100 g. Arterial spin labeling (ASL) is a non-contrast MRI technique that uses water molecules in arterial blood as an endogenous tracer, making it particularly suitable for assessing placental perfusion during pregnancy (9). Diffusion-weighted imaging (DWI), in contrast, reflects the random Brownian motion of water molecules within tissues and is commonly summarized by the apparent diffusion coefficient (ADC) (10).

Although several studies have evaluated placental diffusion and perfusion using 1.5-Tesla MRI, data acquired at 3 Tesla remain limited (11–16). Recent reviews, including that of Manganaro et al. (17), have highlighted that most fetal MRI research has been conducted at 1.5 T, with relatively few studies performed at higher field strengths. The present study therefore contributes to the limited literature on placental MRI at 3T, with particular emphasis on quantitative perfusion measurement in physiological units.

Accordingly, this study aimed to assess placental ADC and perfusion values in pregnancies complicated by IUGR using 3-Tesla MRI.

## Methods

**Study population**: Prospective data were collected from MRI examinations performed between April 2020 and March 2023. The study was approved by the institutional ethics committee (IEC; NI/20/FEB/74/25), and written informed consent was obtained from all participants.

This case–control study included two groups of pregnant women: an IUGR group and a control group. Each group consisted of 30 fetuses, resulting in a total sample of 60 participants. Gestational ages ranged from 20 to 38 weeks. Inclusion and exclusion criteria for both groups are detailed below.

**Figure 1:**
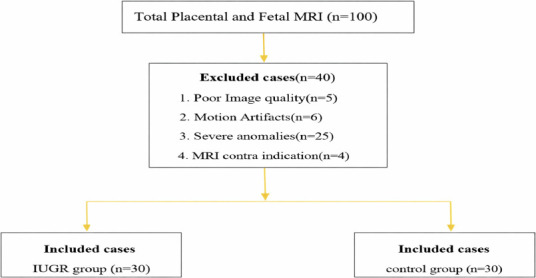
A flowchart illustrating the participant selection process

**Inclusion criteria**: In the IUGR group, fetuses were identified using Hadlock growth curves, with an estimated fetal weight below the 10th percentile for gestational age. Further stratification of IUGR severity was performed using Doppler ultrasonography and included the following criteria (Figure 1):

- Umbilical artery pulsatility index above the 95th percentile
- Elevated umbilical artery pulsatility index combined with a middle cerebral artery pulsatility index below the 5th percentile, suggestive of a brain-sparing effect
- Absent or reversed end-diastolic flow in the umbilical artery
- Absence or reversal of the a-wave in the ductus venosus and/or pulsatile flow in the umbilical vein

Control-group participants were initially referred for MRI because of suspicious ultrasonographic findings; however, subsequent MRI and neonatal evaluations confirmed the absence of abnormalities, justifying their inclusion as normal comparators.

**Exclusion criteria:** Pregnancies with severe fetal anomalies, poor image quality or significant motion artifacts, and contraindications to MRI (including pacemakers or claustrophobia) were excluded (Figure 1).

### Follow-up

Follow-up information was obtained from delivery records and neonatal examination notes in the hospital database. Additional confirmation of neonatal outcomes was obtained through telephone interviews with parents (Figure 1).

**MR imaging**: Imaging was performed on a 3-Tesla MRI scanner using a standardized protocol optimized for placental structure and function. The protocol included anatomical, diffusion-weighted, and perfusion imaging sequences (Table 1). For perfusion analysis, pseudo-continuous arterial spin labeling (pCASL) was performed with labeling at the descending aorta to specifically measure placental perfusion. Respiratory gating with a navigator technique was used to minimize motion artifacts during image acquisition.

**Table 1:** MRI acquisition parameters

Sequence	Plane	TR (ms)	TE (ms)	Matrix	Slice Thickness (mm)	FOV (cm)	NEX	Additional Notes
T2-weighted (SSFSE)	Axial, Sagittal, Coronal	1500–1800	130	512 × 512	5	20	1	Standard anatomical imaging
T1-weighted (FSPGR)	Axial	100–150	2–5	512 × 512	5	20	1	For structural evaluation
Diffusion-Weighted Imaging	Axial	2000–2500	80	256 × 256	4	20	3	b-values: 0 and 700 s/mm^2^; Acquisition ∼1.2 minutes
pCASL Perfusion Imaging	Axial	5000	50	103 × 103	4	20	30	Post-labeling delay: 1525 ms; Time ∼5 min

Acquisition parameters included repetition time (TR), echo time (TE), number of signal averages (NSA), field of view (FOV), slice thickness, and b-values for diffusion-weighted imaging (0 and 700 s/mm^2^). For 3D pCASL imaging, a post-labeling delay of 1525 ms was used, with 30 label–control pairs acquired.

**Post-processing of placental perfusion and diffusion**: Post-processing was performed on a dedicated workstation using Ready View software (AWS), with automated generation of quantitative placental perfusion maps and ADC maps.

**Figure 2:**
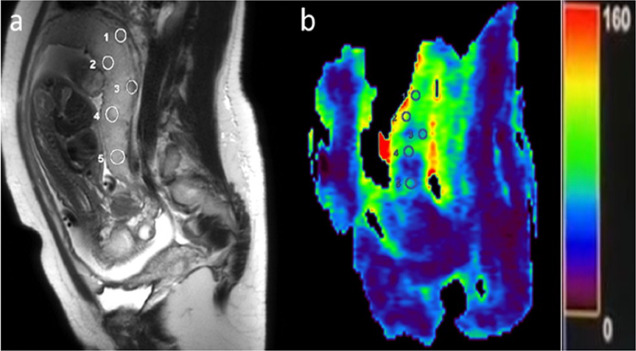
A 25-year-old female with 30 weeks of gestation was referred for placental magnetic resonance imaging (MRI) to evaluate intrauterine growth restriction (IUGR). The MRI revealed a placental blood flow value of 77 ml/min/100 g. (a) T2-weighted SSFSE sagittal image showing the placenta, (b) Placental Pattern-based Morphometry (PBM) map with the Region of Interest (ROI) marked on the placental image

**Perfusion analysis**: Elliptical ROIs measuring 40–60 mm^2^ were manually placed across multiple placental regions on ASL-derived perfusion maps, with reference to T2-weighted anatomical images (Figure 2). Mean placental perfusion for each subject was calculated by averaging all ROI measurements.

**Figure 3:**
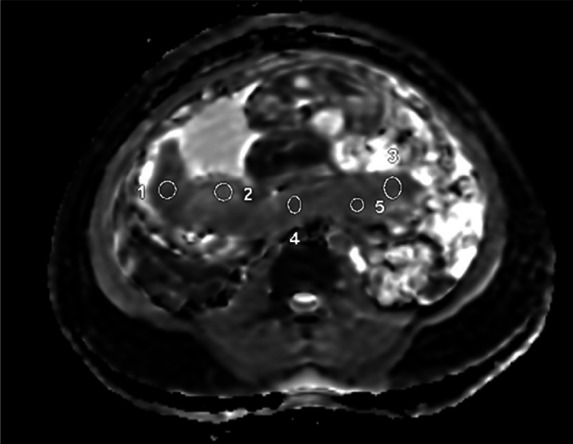
A 25-year-old female at 30 weeks of gestation was referred for placental magnetic resonance imaging (MRI) to evaluate intrauterine growth restriction (IUGR). The MRI revealed an ADC value of 1.734 (10^−3^ mm^2^/s). The ADC map shows the localization of the Region of Interest (ROI) within the placenta

**Diffusion analysis:** ADC values were obtained by placing ROIs in both central and peripheral placental regions across all relevant image slices (Figure 3). Initial ROI placement was performed by a radiologist specializing in fetal MRI. To assess measurement reliability, a second radiologist independently reviewed and repeated measurements in both the control and IUGR groups.

**Statistical analysis**: Statistical analysis was performed using SPSS version 19.0 (IBM Corp., Armonk, NY). Descriptive statistics were used to summarize group-wise means and standard deviations for ADC and perfusion values. Comparisons between IUGR and control groups were conducted using independent-samples t-tests. Inter-observer agreement was assessed using the intraclass correlation coefficient (ICC). ROC curve analysis was performed to evaluate diagnostic performance, including area under the curve (AUC), sensitivity, and specificity. ICC values were interpreted as follows: poor (≤ 0.2), fair (> 0.2–0.4), moderate (> 0.4–0.6), good (>0.6–0.8), and excellent (> 0.8). A p-value ≤ 0.05 was considered statistically significant.

## Results

The study population comprised 60 pregnant women, with 30 participants in each group. Gestational ages ranged from 20 to 38 weeks, with a mean of 26.5 ± 5.2 weeks. Regarding parity, 67% of women were Primigravida, 25% had one previous delivery, and 8% were multiparous. Estimated fetal weights in the study population ranged from 2.5 to 3.4 kg.

**Figure 4:**
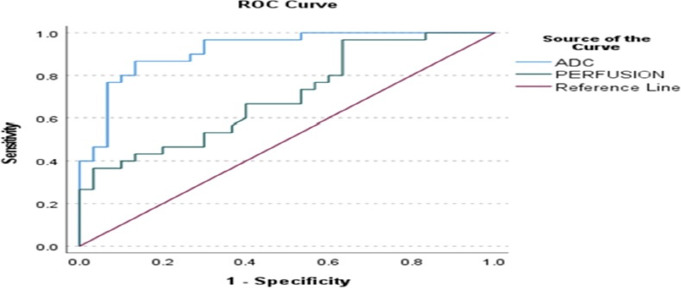
ROC Curve for ADC and Perfusion of the Placenta in IUGR and Control Group

**Perfusion values**: Placental perfusion was significantly reduced in the IUGR group compared with controls (102.5 ± 18.7 vs. 120.2 ± 23.7 ml/100 g/min, p = 0.002) (Table 2). ROC analysis yielded an AUC of 0.703, indicating fair diagnostic accuracy. Using a cut-off value of 93.75 ml/100 g/min, perfusion measurements demonstrated a sensitivity of 86.7% and a specificity of 63.3% (Table 3) (Figure 4).

**Table 2:** Mean and Standard Deviation of ADC and perfusion values of the Placenta along with F and P-values

PARAMETERS		N	Mean ± Std. Deviation	F-Value	P-Value
ADC (10–3 mm2/s)	Controls	30	2.02±0.101	51.708	0.000
	Cases (IUGR)	30	1.83±0.103		
PERFUSION (ml/100g/min)	Controls	30	120.2±23.7		
	Cases (IUGR)	30	102.5±8.7	10.243	0.002

**Table 3:** Showing results of the ROC curve along with sensitivity and specificity values

PARAMETERS	AUC	CUT OFF	SENSITIVITY	SPECIFICITY
ADC	0.919	1.832	96.7	53.3
PERFUSION	0.703	93.75	86.7	63.30

**Apparent diffusion coefficient values**: Mean placental ADC was significantly lower in the IUGR group than in controls (1.83 ±0.10 × 10^−3^ mm^2^/s vs. 2.02 ± 0.10 × 10^−3^ mm^2^/s, p = 0.001) (Table 2). ADC measurements showed excellent diagnostic performance, with an AUC of 0.919. At a cut-off value of 1.832 × 10^−3^ mm^2^/s, ADC demonstrated a sensitivity of 96.7% and a specificity of 53.3% (Table 3) (Figure 4).

**Inter-observer agreement**: Inter-observer reproducibility was excellent, with an ICC value of 0.8 for both ADC and perfusion measurements.

## Discussion

This study evaluated placental ADC and perfusion values in pregnancies complicated by IUGR using 3T MRI in an Indian population. Significant reductions in both parameters were observed in the IUGR group compared with controls, reflecting impaired placental function.

Mean ADC values were significantly lower in IUGR placentas (1.83 ± 0.103 × 10^−3^ mm^2^/s) than in controls (2.02 ± 0.101 × 10^−3^ mm^2^/s). These findings are consistent with those reported by Razek et al. (13), who observed reduced ADC values in IUGR using 1.5T MRI. Slight differences in absolute values may reflect variations in field strength, spatial resolution, and population characteristics. Similar trends have been reported by Görkem et al. (8), further supporting the association between reduced ADC and placental dysfunction in IUGR.

ROC analysis in the present study demonstrated excellent diagnostic performance for ADC (AUC = 0.919), with higher sensitivity but lower specificity compared with previous studies (8,13). This variability underscores the influence of imaging protocols and population characteristics on diagnostic thresholds.

Placental perfusion was also significantly reduced in IUGR pregnancies, consistent with prior ASL-based studies (14,15). Although absolute perfusion values differed across studies—likely due to differences in ASL technique and field strength—the relative reduction in perfusion in IUGR placentas was a consistent finding.

It should be noted that ADC values in highly perfused tissues incorporate a perfusion component (“perfusion bias”). Consequently, the observed reduction in ADC in IUGR placentas is at least partly attributable to decreased perfusion. For this reason, IVIM models that separate diffusion and perfusion components are increasingly used in placental imaging. Our findings of reduced perfusion using 3D pCASL are consistent with IVIM-based studies reporting decreased perfusion fractions in IUGR (18). Importantly, pCASL enables direct quantification of perfusion in absolute physiological units, enhancing the clinical and translational relevance of these measurements.

This study has several limitations. First, the sample size was relatively small. Second, despite the use of respiratory gating, residual maternal and fetal motion may have affected image quality. Larger prospective studies are warranted to validate these findings.

In conclusion, in this pilot study, both placental ADC and perfusion values were significantly reduced in pregnancies complicated by IUGR. Given the influence of perfusion bias on ADC measurements, the most clinically relevant finding is the direct quantification of reduced placental perfusion using 3D pCASL at 3T. This technique provides absolute perfusion values in physiological units and reinforces the potential role of advanced MRI methods—particularly pCASL—in the non-invasive assessment of placental function and early detection of IUGR.
